# MoO_3_/S@g-C_3_N_4_ Nanocomposite Structures: Synthesis, Characterization, and Hydrogen Catalytic Performance

**DOI:** 10.3390/nano13050820

**Published:** 2023-02-23

**Authors:** Alhulw H. Alshammari, Majed Alshammari, Sultan Alhassan, Khulaif Alshammari, Turki Alotaibi, Taha Abdel Mohaymen Taha

**Affiliations:** Physics Department, College of Science, Jouf University, Sakaka P.O. Box 2014, Saudi Arabia

**Keywords:** hydrogen catalyst, MoO_3_, S@g-C_3_N_4_ nanosheet, nanocomposite, NaBH_4_, band gap energy

## Abstract

Hydrogen production as a source of clean energy is high in demand nowadays to avoid environmental issues originating from the use of conventional energy sources i.e., fossil fuels. In this work and for the first time, MoO_3_/S@g-C_3_N_4_ nanocomposite is functionalized for hydrogen production. Sulfur@graphitic carbon nitride (S@g-C_3_N_4_)-based catalysis is prepared via thermal condensation of thiourea. The MoO_3_, S@g-C_3_N_4_, and MoO_3_/S@g-C_3_N_4_ nanocomposites were characterized using X-ray diffraction (XRD), Fourier transform infrared spectroscopy (FTIR), Field Emission Scanning Electron Microscope (FESEM), STEM, and spectrophotometer. The lattice constant (a = 3.96, b = 13.92 Å) and the volume (203.4 Å^3^) of MoO_3_/10%S@g-C_3_N_4_ were found to be the highest compared with MoO_3_, MoO_3_/20-%S@g-C_3_N_4_, and MoO_3_/30%S@g-C_3_N_4_, and that led to highest band gap energy of 4.14 eV. The nanocomposite sample MoO_3_/10%S@g-C_3_N_4_ showed a higher surface area (22 m^2^/g) and large pore volume (0.11 cm^3^/g). The average nanocrystal size and microstrain for MoO_3_/10%S@g-C_3_N_4_ were found to be 23 nm and −0.042, respectively. The highest hydrogen production from NaBH_4_ hydrolysis ~22,340 mL/g·min was obtained from MoO_3_/10%S@g-C_3_N_4_ nanocomposites, while 18,421 mL/g·min was obtained from pure MoO_3_. Hydrogen production was increased when increasing the masses of MoO_3_/10%S@g-C_3_N_4_.

## 1. Introduction

The use of conventional energy sources, i.e., fossil fuels, has led to the global climate change crisis, which has become a serious environmental issue [1,2]. The negative impact of using such energy sources resides in their emissions of toxic gases such as NO_X_, CO_X_, and SO_X_. Exposure to these gases at certain concentrations may cause serious health issues in humans and animals [3,4,5]. Therefore, people have been stimulated to use renewable energy sources like solar cells and hydrogen production, aiming to maintain the stability of the environment [1,2,6]. Solar cells have been used for the conversion of sunlight to power. The efficiency of solar cells is limited to the condition of the weather, hence, the solar cell produces more power on a sunny day compared with a cloudy day. In addition, the power generated by the solar cells must be stored in batteries to be used whenever needed. Economically, generating power from solar cells is not favorable due to their low efficiency and high cost of energy storage. Therefore, hydrogen production has superior proprieties to solar cells, and so it becomes a hotspot topic nowadays. Hydrogen exists in nature as an abundant element i.e., water [7].

Nanomaterials have a significantly large surface area to volume ratio due to their small dimensions. The surface properties of nanomaterials will have an impact on the entire material, particularly when their sizes are comparable in terms of length [2,6]. Thus, the properties of the bulk materials can be improved upon or modified. Since a pioneer study that was published in 1972 about the use of TiO_2_ electrodes for water splitting into hydrogen [8], many photocatalytic studies have been conducted for hydrogen production using nanomaterials i.e., metal oxides, carbon nitride, nanosheets doped with sulfur, graphitic carbon nitride (GCN), conjugated polymers, etc. [9,10,11]. Graphitic carbon nitride is a layered substance made of carbon and nitrogen atoms. Simple thermal condensation of urea, thiourea, and melamine into anodic alumina templates is used to synthesize GCN nanostructures. The high thermal stability of the GCN structures extends to 600 °C and these structures are highly chemically stable when exposed to acid, base, and organic solvent attacks. The band gap of these materials is approximately 2.7 eV [12]. Additionally, these materials are promising materials for fuel cells, photocatalysis, heterogeneous catalysis, light emitting devices, and surface modification [12,13].

There are several study reports on the preparatory, structural, optical, and many other characteristics of metal nanocomposites, as well as their applications in various fields. Building nanocomposite structures with distinctive morphologies and large surface areas is a frequent structural engineering technique for presenting more active sites.

Material composites such as Molybdenum disulfide MoS_2_/graphene as co-catalytic to TiO_2_ have achieved a high rate of hydrogen production ~165.3 μmol h^−1^ [14]. Molybdenum Oxide (MoO_3_) is known to be environmentally friendly, inexpensive, and own high activity, therefore it has been extensively used for electrocatalysis [1,15,16]. On other hand, the use of MoO_3_ for hydrogen production has limitations during HER electrocatalyst because of its strong Molybdenum and Hydrogen (Mo-H) bond, which highly resists hydrogen adsorption and indeed has a negative impact on hydrogen production [1,17,18]. Therefore, developing new strategies to decrease the adsorption of hydrogen on the sites of Mo metal is highly needed to improve the MoO_3_ catalytic process. Recently, various methods have been conducted to resolve the issue of catalytic activity when producing a robust electrode, for instance, the design of interface structure, defect engineering, and doping metal with anion and cation [19,20,21,22,23]. Doping the composition with suitable ions is the most effective and common method because it leads to structural improvement. Therefore, doping the MoO_3_ with anion elements can reduce its density states and lead to weak Mo-H bonding during the HER electrocatalyst [24,25]. Moreover, doping the MoO_3_ with cations has enhanced its electronic structure, that it led to the kinetics expedition of the HER catalytic reaction. The catalytic activity and the surface area of the MoO_3_ nanosheet in the alkaline medium, when doped with copper (Cu) atom, have been enhanced [1]. In addition, the electron–hole recombination of MoO_3_ typically leads to low photocatalytic efficiency, therefore coating the MoO_3_ with suitable materials delays the electron–hole recombination. Recently, semiconducting materials like g-C_3_N_4_ have been used to overcome the photocatalytic issues for other nanocomposites. For example, g-C_3_N_4_/TaON [26], g-C_3_N_4_/ZnO [27], and g-C_3_N_4_/Bi_2_WO_6_ [28] because it has remarkable photocatalytic performance [29,30]. The g-C_3_N_4_ is a soft polymeric semiconductor, which can be readily coated on other nanocomposites.

Therefore, in this work, the MoO_3_/S@g-C_3_N_4_ is functionalized for hydrogen production. When S@g-C_3_N_4_ and MoO_3_ were combined, the S@g-C_3_N_4_ was coated on the surface of MoO_3_ and turned to be positively charged. In addition to that, the combination of MoO_3_ and S@g-C_3_N_4_ has led to a large band gap energy of ~4.14 eV. These outcomes delayed electron–hole recombination, which enhanced its photocatalytic performance and increased hydrogen production. The highest hydrogen production from NaBH_4_ hydrolysis ~22,340 mL/g·min was obtained from MoO_3_/10-S@g-C_3_N_4_ nanocomposites while 18,421 mL/g·min was obtained from pure MoO_3_.

## 2. Experimental

### 2.1. Chemicals and Reagents

Thiourea and extra pure sodium borohydride were provided from Loba Chemie, Mumbai, India. MoO_3_ nanopowder, ethanol absolute, and methanol absolute were supplied from Sigma-Aldrich, Darmstadt, Germany.

### 2.2. Nanocomposite Preparation

Sulfur@carbon nitride nanosheet was prepared via thermal condensation. 15 g of thiourea are inserted into a porcelain crucible and heated at 550 °C for 2 h at a heating rate of 3.0 °C/min. The crucible was taken from the furnace and the yellow powder was washed with distilled water.

MoO_3_/S@g-C_3_N_4_ catalyst nanocomposites were prepared with different proportions of carbon nitride. The powders of MoO_3_ (90, 80, 70 wt%) and S@g-C_3_N_4_ (10, 20 and 30 wt%) were mixed in ethanol for 1 h on a magnetic stirrer at 300 K. Then, the solution was subjected to an ultrasonic bath for 1 h. After that, the solution was dried at 100 °C overnight. The obtained powder was ground very well for 30 min.

### 2.3. Characterization of Nanocomposite

An effective method for determining the crystalline structure based on the interaction of materials and electromagnetic radiation is the XRD analysis. The data of XRD spectra were conducted using a Shimadzu diffractometer (XRD 7000, Kyoto, Japan). Software can be employed to index the crystal structures of samples by comparing the obtained XRD patterns to the crystalline database. Identification of the functional groups in the material can be completed with the use of FTIR spectroscopy. The ATR spectra were obtained using a Shimadzu spectrometer (FTIR–Tracer 100, Kyoto, Japan). The scanning electron microscope is a tool for the morphological analysis of materials that scans the surface with a focused electron beam. FESEM micrographs were recorded on the Quattro ESEM’s environmental scanning electron microscope (Thermo Fisher Scientific, Waltham, MA, United States). The samples were placed on carbon tape and their surface was coated with gold. STEM microscopy analysis was completed on a Talos F200i TEM/STEM electron microscope (Thermo Fisher Scientific, Waltham, MA, USA). In order to investigate the physical characteristics of porous materials, such as specific surface area, pore size distribution, and pore volume, we have completed N_2_ sorption/desorption analysis. NOVA 4200e chemo-physisorption surface area analyzer was used to get the conventional N_2_ adsorption isotherms point-by-point by measuring the quantity of nitrogen adsorbed and the equilibrium pressure at 77 K. The samples were initially outgassed for a continuous 24 h at 150 °C and 1 millitorr vacuum. The sample is cleaned and ready for the collection of adsorption data after this outgassing procedure. The UV-Vis analysis is an important technique for determining a semiconductor band gap and for analyzing a photocatalyst capacity for absorption. The optical absorption spectroscopy data were conducted on a Thermo Scientific Evolution 200 UV-Vis spectrophotometer (Waltham, MA, USA). The samples were dissolved in distilled water via ultrasonic waves to get a suspension.

### 2.4. Hydrogen Catalytic Performance

Catalytic hydrogen evolution at room temperature was used to assess the catalytic activity of the prepared composites. The hydrogen catalytic tests were completed in a 250 mL conical flask with 0.25 g of NaBH_4_ solution that was hydrolyzed with 10.0 mL methanol. The reaction temperature for the system was constant as the glass flask was placed in a water bath. No stirring was employed in the reaction flask. The temperature of the water bath was kept constant at 25 °C. The masses of catalyst and NaBH_4_ were mixed very well and then inserted into the conical flask. After that, 10 mL methanol was added to the glass flask and the stopwatch started simultaneously. The volume of generated hydrogen gas was measured by measuring the displacement of the water level in the burette.

## 3. Results and Discussion

### 3.1. XRD Analysis

**Figure 1:**
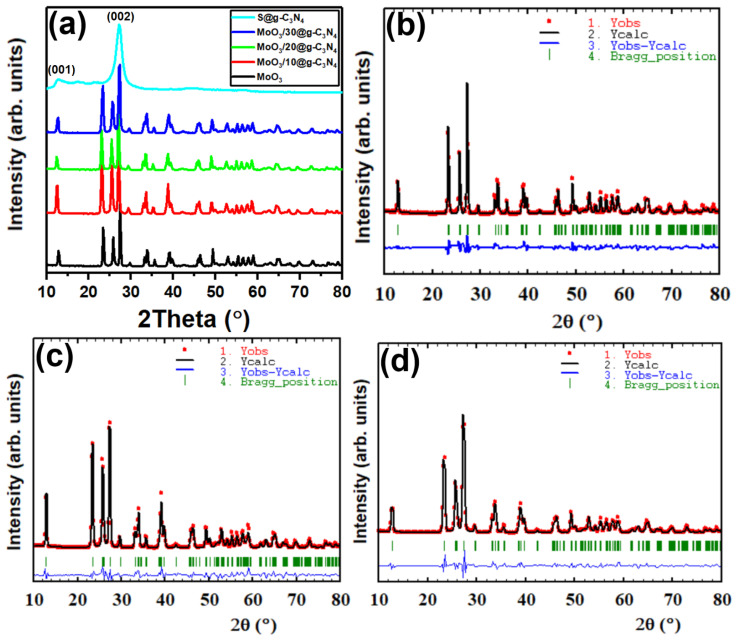
(**a**) XRD diffraction patterns of graphitic carbon nitride S@g-C_3_N_4_, MoO_3_, and MoO_3_/S@g-C_3_N_4_ nanocomposites with varying g-C_3_N_4_ concentrations. (**b**–**d**) Rietveld refinement of XRD patterns of MoO_3_, MoO_3_/10%S@g-C_3_N_4_, and MoO_3_/30%S@g-C_3_N_4_, respectively.

The XRD patterns’ peaks of nanocomposites MoO_3_, S@g-C_3_N_4_, and MoO_3_/S@g-C_3_N_4_ are shown in Figure 1a. The diffraction peaks correspond to (020), (110), (021), (120), (400), (060), and (622) planes with peak positions at 2*θ* = 12.8°, 23.4°, 25.6°, 27.4°, 33.8°, 39.2°, and 49.4°, respectively. All of the peaks of MoO_3_ belong to data of the orthorhombic structure according to JCPDF card number (35-0609) [31]. The average nanocrystal size of MoO_3_/S@g-C_3_N_4_ was calculated as shown in Table 1, using the Scherer equation [32,33,34]:

(1) $$D=\frac{0.9\lambda }{\beta \cos\theta }$$

where λ is the X-ray radiation of wavelength and β is the full width at half maximum.

The S@g-C_3_N_4_ diffraction peaks at 27.5° and 13.02° correspond to the (002) and (100) planes, which is consistent with the interplanar staking peaks characteristic of aromatic systems and the inter-layer structure packing, respectively [35]. In the case of MoO_3_/S@g-C_3_N_4_ composites, however, the S@g-C_3_N_4_ diffraction peaks are not clearly recognized. This outcome indicates that S@g-C_3_N_4_ nanosheets coat the surface of the MoO_3_ nanocrystal. All of the diffraction peaks of MoO_3_/S@g-C_3_N_4_ composites are observed to shift from 27.2° to 27.4° with different S@g-C_3_N_4_ concentrations, and this is a result of the two lines overlapping. Consequently, the different concentration of S@g-C_3_N_4_ in the expanded XRD diffraction pattern confirms the coexistence of MoO_3_ and g-C_3_N_4_ in the MoO_3_/S@g-C_3_N_4_ composites. Moreover, the less-ordered crystalline structure of S@g-C_3_N_4_ is expected to show an intense thermal etching process with many defects, which is intended to improve catalytic activity.

The plots obtained by the Rietveld refinement of MoO_3_, MoO_3_/10%S@g-C_3_N_4_, MoO_3_/20S@g-C_3_N_4_, and MoO_3_/30S@g-C_3_N_4_ are shown in Figure 1b–d. Crystallography open database (COD) was used to find matching reference patterns, which were used to set the initial values for space group, cell parameters, and atom coordinates. The background was improved by applying the cosine Fourier series with six different coefficients that could be modified, and the Bragg reflection profile was characterized by the Thompson-Cox-Hastings pseudo-Voigt function. Several factors were refined, including unit cell parameters, scale factor, structure factor, occupancy, position parameters, etc. The observed and calculated diffractograms are in good agreement for both types of synthesis processes, as shown in Figure 1c,d. Also, Table 1 provides an overview of the comparison of lattice parameters and quality of fit. It is crucial to note that there is a change in lattice parameters for the MoO_3_/10%S@g-C_3_N_4_ sample when compared to other concentrations of wt% S@g-C_3_N_4_; an increase in lattice constants that can be ascribed to the S@g-C_3_N_4_ doping on MoO_3_ structure. In particular, an increase in the value of the lattice constant “b” was found at 10 wt% MoO_3_/S@g-C_3_N_4_ concentration and a decrease in the lattice parameter “b” was observed for samples containing 20 wt% and 30 wt% of S@g-C_3_N_4_. The decrease in the lattice parameter “b” suggests that the MoO_3_ crystal structure is under compression [36]. Previous work has shown that the lattice parameter can be increased due to the oxygen vacancies, and that might be decreased by raising the annealing temperature, which decreases oxygen vacancies and enhances crystallinity [37,38,39].

### 3.2. FTIR Structure Analysis

**Figure 2:**
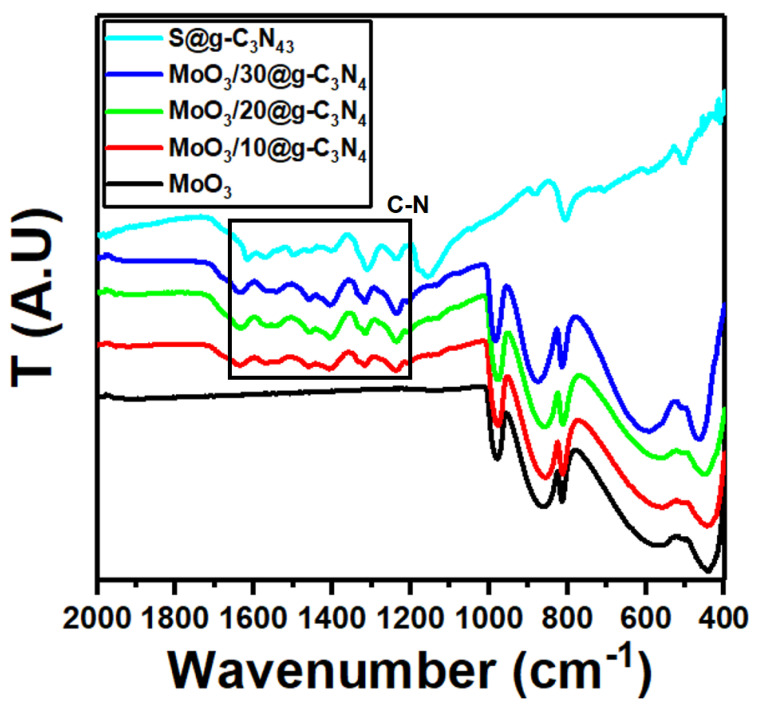
FTIR spectra of MoO_3_, S@g-C_3_N_4_, and MoO_3_/S@g-C_3_N_4_ nanocomposites with different concentrations of S@g-C_3_N_4_.

The FT-IR spectra of MoO_3_, S@g-C_3_N_4_, and different concentrations of MoO_3_/S@g-C_3_N_4_ composites are shown in Figure 2. The peak at 1643 cm^−1^ for pure S@g-C_3_N_4_ is attributable to C=N stretching vibration modes, while the peaks at 1242, 1322, 1405 cm^−1^, and 1563 cm^−1^ are correlated with aromatic C-N stretching [40,41]. The band at 809 cm^−1^ corresponds to the C-N heterocycles with out-of-plane bending modes [42]. The vibrations in pure MoO_3_ appeared at approximately 561, 866, and 990 cm^−1^, which are formed by the oxygen stretching mode associated with three metal atoms, the oxygen stretching mode in the Mo-O-Mo units, and the Mo=O stretching mode, respectively [43,44]. The characteristic vibrations for MoO_3_ and S@g-C_3_N_4_ persist in MoO_3_/S@g-C_3_N_4_ composites and the absorption bands in MoO_3_/S@g-C_3_N_4_ slightly enhanced as the S@g-C_3_N_4_ concentration increases. These outcomes agree with the XRD structural data.

### 3.3. ESEM and STEM Microscopy

**Figure 3:**
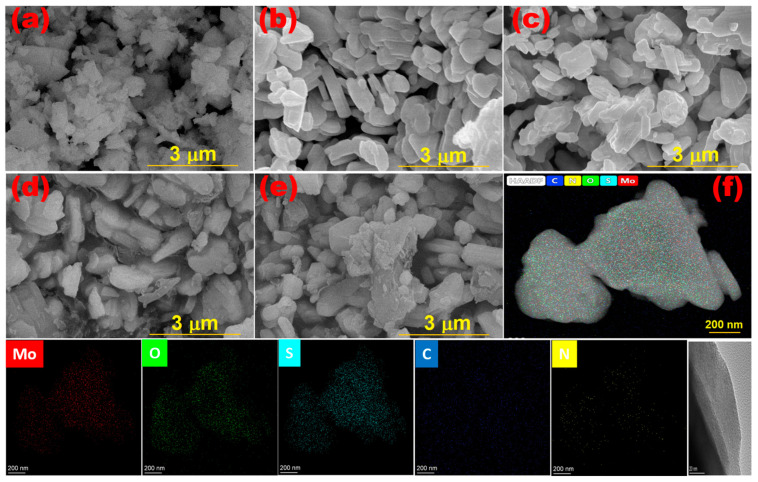
Morphological images of (**a**) S@g-C_3_N_4_, (**b**) MoO_3_, (**c**) MoO_3_/10%S@g-C_3_N_4_, (**d**) MoO_3_/20%S@g-C_3_N_4_, (**e**) MoO_3_/30%S@g-C_3_N_4_ and (**f**) STEM image of MoO_3_/10%S@g-C_3_N_4_.

The morphology of materials composites enhances the understanding of their microstructure and contributes to the identification of suitable applications. Therefore, the surfaces of the MoO_3_/S@g-C_3_N_4_ nanocomposites were scanned using the FESEM microscope and the images were collected in Figure 3a–e. The S@g-C_3_N_4_ shown in Figure 3a takes the form of scattered flakes because of the sticky layers [45]. While the micrograph for MoO_3_ nanocrystals showed orthorhombic shapes (Figure 3b). In the images of MoO_3_/S@g-C_3_N_4_ nanocomposites (Figure 3c–e), MoO_3_ crystals appear wrapped with thin random flakes of carbon nitride. Moreover, the thickness of the S@g-C_3_N_4_ layers increases with increasing content from 10 to 30%. These observations are evidence of the strong interaction between MoO_3_ and S@g-C_3_N_4_.

In Figure 3f, a STEM image of the MoO_3_/10%S@g-C_3_N_4_ was shown and the formation of the sticky flakes coating the surface of MoO_3_ was confirmed. It also showed the mapping of Mo, oxygen, sulfur, carbon, and nitrogen atoms. The distribution of elements is homogeneous and thus confirms the formation of the nanocomposite. Moreover, the STEM image confirms the scans of SEM.

### 3.4. Surface Area and Pore Size 

**Figure 4:**
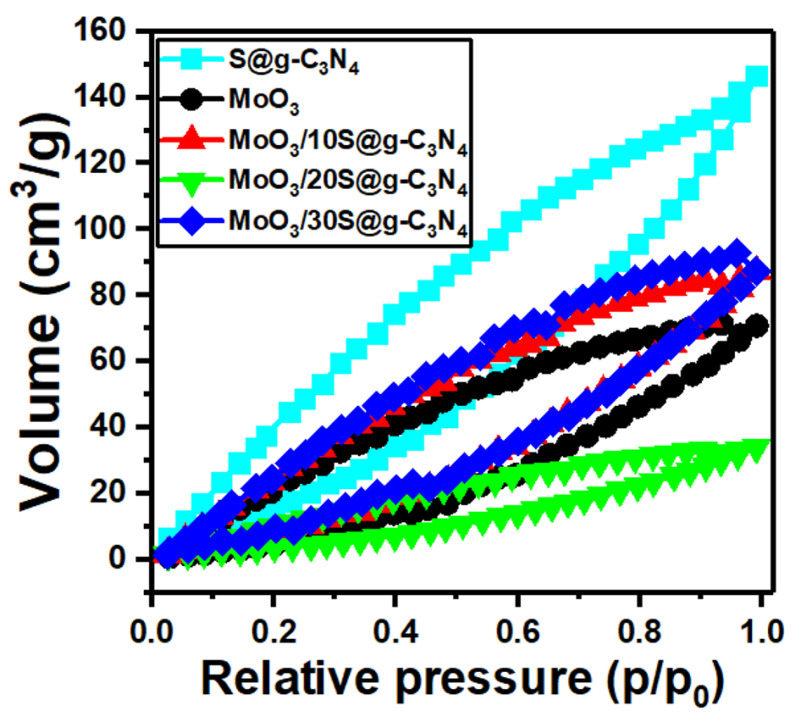
N_2_ adsorption−desorption plots of MoO_3_/10%S@g-C_3_N_4_ samples.

The adsorption−desorption isotherms for the MoO_3_/S@g-C_3_N_4_ nanocomposites are displayed in Figure 4. These isotherms belong to type IV mesoporous materials. The surface area of samples is often determined using a traditional Brunauer-Emmet-Teller (BET) model. Moreover, the surface area BET for these nanocomposites was determined. The surface area values are 40.0, 19.0, 22, 9.0, and 2.5 m^2^/g for the samples S@g-C_3_N_4_, MoO_3_, MoO_3_/10%S@g-C_3_N_4_, MoO_3_/20%S@g-C_3_N_4_, and MoO_3_/30%S@g-C_3_N_4_, respectively. 

The nanocomposite sample MoO_3_/10%S@g-C_3_N_4_ revealed a higher surface area. The increased surface area promotes the reaction rate simply by introducing more active sites to the reactants. The BJH pore volume data were 0.18, 0.1, 0.11, 0.04, and 0.01 cm^3^/g for the samples S@g-C_3_N_4_, MoO_3_, MoO_3_/10%S@g-C_3_N_4_, MoO_3_/20%S@g-C_3_N_4_, and MoO_3_/30%S@g-C_3_N_4_, respectively. Accordingly, the nanocomposite sample MoO_3_/10%S@g-C_3_N_4_ showed a higher surface area and large pore volume. Large pore volumes work in a different way, in that larger pore volumes can offer more interior voids as nano-reactors to physically confine the reactants in specific areas, enhance active species concentrations, and therefore dynamically promote mass transfer. Therefore, this sample contains more active sites and has a high adsorption capacity.

### 3.5. UV-Vis Spectroscopy

**Figure 5:**
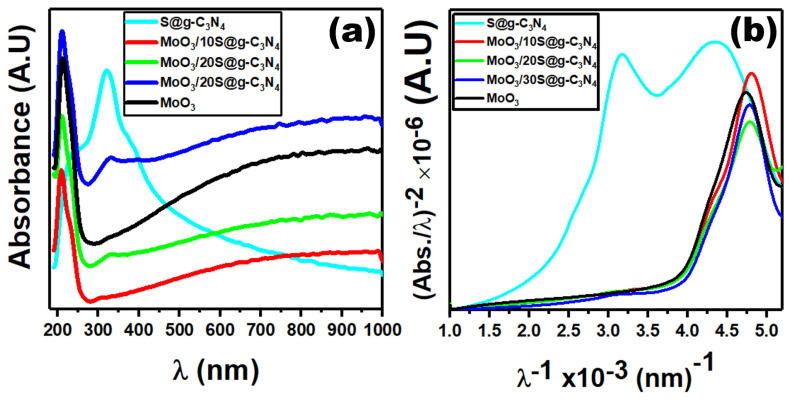
Plots of (**a**) optical absorption and (**b**) ASF graphs for nanostructures.

The optical measurements aim to calculate the optical band gap of S@g-C_3_N_4_, MoO_3_/10%S@g-C_3_N_4_, MoO_3_/20%S@g-C_3_N_4_, MoO_3_/30%S@g-C_3_N_4_, and MoO_3_ nanocomposites. We demonstrated optical absorption spectra to elucidate the optical properties using a spectrophotometer. Due to n→π* electronic transitions, the absorbance spectra exhibit a high absorption peak centered at 322 nm. We use Tauc’s plot and the ASF formula to determine the optical band gap. For crystalline materials, the following equations are used to study the absorption coefficient and incident photon energy [46,47]:

(2) $$\alpha \;\left(v\right)hv=k{\left(hv-E_{gab}\right)}^{r}$$

(3) $$\alpha \;\left(v\right)=k{\left(hc\right)}^{r-1}{\left(\frac{1}{\lambda }-\frac{1}{\lambda_{gab}}\right)}^{r}$$

where α (v) is the absorption coefficient, $E_{gab}$ is the optical gap, hv represents incident photon energy, k is a constant, r is the optical charge carrier direct transition index which is equal to 1/2. $\lambda_{gab}$, $E_{gab}$ represents the optical gap ($E_{gab}\left(eV\right)=\frac{1239.83}{\lambda_{gab}}$), and h and c are Plank’s constant and the light velocity, respectively. In order to calculate the optical band gap, we use Beer-Lambert’s law. This law is determined by $A\left(\lambda \right)=\left(\frac{\alpha_{1}}{2.303}\right)\alpha_{2}$ where $\alpha_{1}$ and $\alpha_{2}$ are the solution concentration and absorbance, respectively. Thus, Equation (2) becomes [48]:

(4) $$A\left(\lambda \right)=D\lambda {\left(\frac{1}{\lambda }-\frac{1}{\lambda_{gab}}\right)}^{\frac{1}{2}}$$

where D is represented as $D=k\;{\left(hc\right)}^{m-1}\;\frac{\alpha_{1}}{2.303}$. Figure 5 shows the plot of ${\left(\frac{A_{\lambda }}{\lambda }\right)}^{2}$ against $\lambda^{-1}$, where we extrapolate the straight-line portion of this plot at ${\left(\frac{A_{\lambda }}{\lambda }\right)}^{2}=0$. Then, we determined the $E_{gap}^{ASF}$ value, which was 2.4 eV for S@g-C_3_N_4_ nanosheet. Moreover, the energy gaps of MoO_3_, MoO_3_/10%S@g-C_3_N_4_, MoO_3_/20%S@g-C_3_N_4_, and MoO_3_/30%S@g-C_3_N_4_ nanostructures were 3.86, 4.14, 4.0, and 4.12 eV, respectively. The energy gap for MoO_3_/10%S@g-C_3_N_4_ is that of MoO_3_, MoO_3_/20%S@g-C_3_N_4_, and MoO_3_/30%S@g-C_3_N_4_. The lattice constant and the volume of 10%S@g-C_3_N_4_, MoO_3_ are increased leading to higher band gap energy [49]. The efficiency of the photocatalyst is enhanced by increasing the band gap energy. The increased band gaps are attributed to the strong quantum effect produced by the ultra-thin atomic-thick S@g-C_3_N_4_ nanosheets. For example, doping CdS, Fe_2_O_3_, and WO_3_ with TiO_2_ improve the optical absorption and charge carrier separations. The larger the band gaps, the greater the reductive capacity, and hence a more favorable thermodynamic driving force for H_2_ generation. The sample (MoO_3_/10%S@g-C_3_N_4_) is expected to improve hydrogen production [50].

Mulliken electronegativity theory was used to predict the location of the CB and VB edges of MoO_3_/S@g-C_3_N_4_. Accordingly, the following formula can be used to estimate the location of the valence band (*E_VB_*) [51]:

(5) $$E_{VB}=\chi -E+0.5E_{g}$$

where *χ* is the MoO_3_ electronegativity (6.4 eV), *E* is electron free energy (4.5 eV), and *E_g_* is the determined band gap [52]. Further, the position of conduction band (*E_CB_*) is estimated according to valence band and the band gaps [53]:

(6) $$E_{CB}=E_{g}-E_{VB}$$

The calculated values of valence band positions are 3.83, 3.97, 3.90, and 3.96 eV for MoO_3_, MoO_3_/10%S@g-C_3_N_4_, MoO_3_/20%S@g-C_3_N_4_, and MoO_3_/30%S@g-C_3_N_4_ nanocomposites. The conduction band energies for the same nanocomposite samples are 0.03, −0.11, −0.04, and −0.1 eV. The activity of a catalyst for each reaction is influenced by changes in the conduction and valence bands caused by coating with S@g-C_3_N_4_.

### 3.6. Hydrogen Catalytic Performance

**Figure 6:**
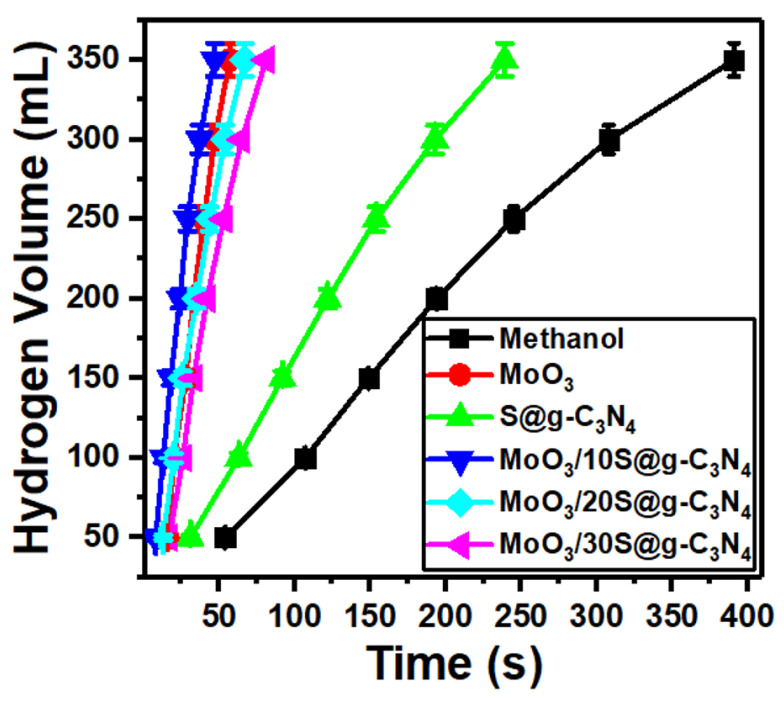
Hydrogen evolution from methanolysis of NaBH_4_ at different MoO_3_/S@g-C_3_N_4_ ratios.

Self-hydrolysis of NaBH_4_ at room temperature generates a very low volume of hydrogen due to an increase in pH during the hydrolysis reaction. The main reason for the pH raise is the induced by-product of strongly basic sodium metaborate (NaBO_2_) ion [54]. Therefore, to implement an H_2_ economy, the development of a catalyst with the ability to cause a high generation rate at room temperature is necessary and essential. High hydrogen generation rates with great control are accomplished via a catalyst. Primary alcohols are used as reactants in the place of water or as a partial substitute for water in a different method of producing hydrogen from sodium borohydride. Methanol is the lightest alcohol and has the highest reactivity toward sodium borohydride, making it an effective alternative to water as a reactant for the generation of hydrogen [55]. The measurements of hydrogen evolution from the reaction of NaBH_4_-methanolysis are shown in Figure 6. 20 mg of catalysts (MoO_3_, S@g-C_3_N_4_, and MoO_3_/S@g-C_3_N_4_) were used to test their efficiency in producing hydrogen. The catalysts connected with the amino group catalyze the NaBH_4_ hydrolysis and methanolysis for the production of hydrogen according to the mechanism of Langmuir-Hinshelwood; as the molecules of methanol and NaBH_4_ adsorbed on the surface of the catalyst [56]. Otherwise, NaBH_4_ can be adsorbed without methanol on the catalyst surface as described by the mechanism of Michaelis-Menten [57]. It can be concluded from the foregoing that the surface properties of the catalyst are of great importance in the production of hydrogen.

In methanol, NaBH_4_ is initially decomposed into Na^+^ and BH_4_ ions. A second stage might be the adsorption of the produced BH_4_ ions onto the charged surface of the MoO_3_/S@g-C_3_N_4_ catalyst. Thus, the large positive area located on the surface of the catalyst will increase the adsorption of BH_4_ ions. 1.0 mole of H_2_ is produced by the interaction of the hydrogen atom with a negative charge in the structure of the catalyst complex-H that is induced because of electron transfer and the hydrogen atom with positive charge of methanol. BH_3_ and the methoxy ion of 3.0 mole of methanol combine simultaneously to generate B(CH_3_O). Finally, 4H_2_ is generated and NaB(OCH_3_)_4_ was produced [56,57]. 

From Figure 6, it can be seen that the maximum amount of hydrogen produced is accelerated with the addition of nanocomposites. In addition, the fastest hydrogen evolution was revealed with the sample MoO_3_/10%S@g-C_3_N_4_. The methanolysis of NaBH_4_ material includes two products of Na^+^ and BH_4_^−^ ions. Thereafter, the developed BH_4_^−^ ions are adsorbed on the charged surface of the MoO_3_/10%S@g-C_3_N_4_. Thus, a catalyst with a high positively-charged surface adsorb BH_4_^−^ ions in short times [58]. Moreover, the fitting of hydrogen volume versus time for S@g-C_3_N_4_, MoO_3_, MoO_3_/10%S@g-C_3_N_4_, MoO_3_/20%S@g-C_3_N_4_, and MoO_3_/30%S@g-C_3_N_4_ nanocomposites give slopes of 1.60, 8.0, 8.71, 6.22, and 4.74, respectively. The nanocomposite sample MoO_3_/10%S@g-C_3_N_4_ showed the highest slope (8.71) and thus possesses the highest hydrogen generation rate. 

**Figure 7:**
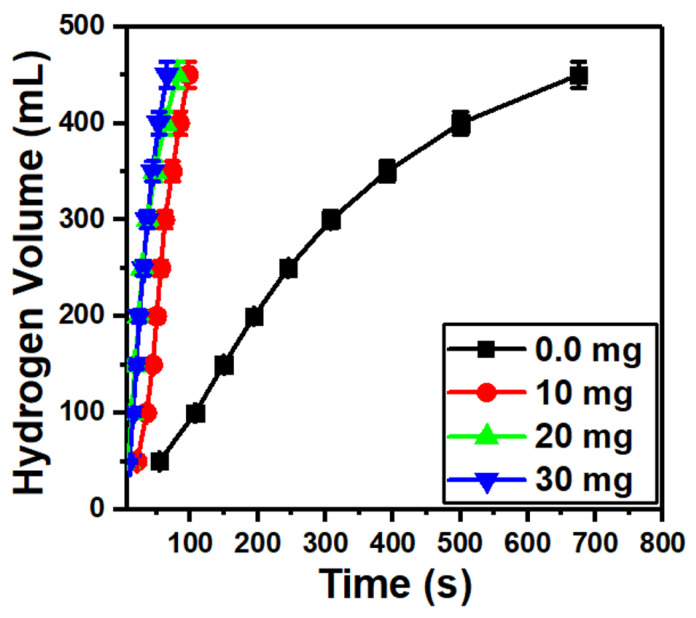
Hydrogen generation from NaBH_4_ hydrolysis at different masses of MoO_3_/10S@g-C_3_N_4_.

Figure 7 shows a fast hydrogen generation as the MoO_3_/10%S@g-C_3_N_4_ loading is increased from 0.0 to 30 mg. This may be due to the wide energy gap of the MoO_3_/10%S@g-C_3_N_4_ catalyst, which possesses a high separation of charge carriers [59]. Moreover, the amino group from S@g-C_3_N_4_ that is coated on the surface of MoO_3_ motivates the hydrogen production from NaBH_4_. 

One of the most crucial factors that must be taken into account while developing engineering solutions for hydrogen energy applications, is hydrogen generating rate. The H_2_ generation rate (*r*) for the MoO_3_/%S@g-C_3_N_4_ catalyst is estimated from the following relation [60,61]:

(7) $$r=\frac{V}{t\cdot m_{cat}}$$

where *V* denotes the H_2_ volume, *m_cat_* is the mass of the catalyst, and *t* is the time of reaction. The H_2_ generation rates that were obtained based on the data in Figure 7, nevertheless, have decreased. As the values of *r* were 28,767, 22,340, and 15,905 mL/g·min at 10, 20, and 30 mg of MoO_3_/10%S@g-C_3_N_4_ catalyst, respectively. A reduction in the catalytic activity of the methanolysis process is the end outcome, which is the blockage of the catalyst active sites as a result of the catalyst active sites becoming saturated [58].

**Figure 8:**
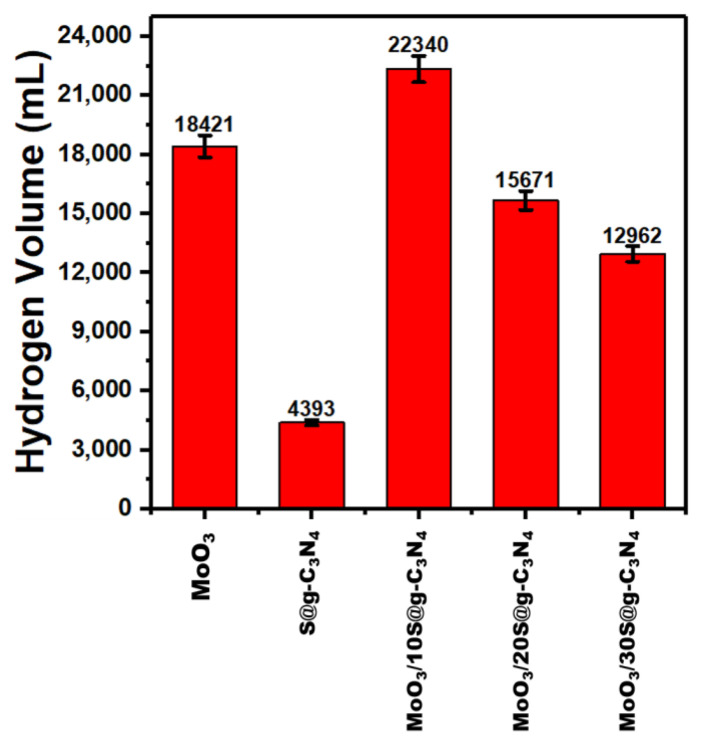
Hydrogen evolution rates at different nanocomposites.

Figure 8 shows the influence of nanocomposite catalysts on the rate of hydrogen generated. The nanocatalyst MoO_3_/10%S@g-C_3_N_4_ achieved a higher generation rate of 22,340 mL/g·min. The higher generation rate of composite comes due to the separation of charge carriers and positively charged areas on the surface of the catalyst.

The comparison of hydrogen evolution rate for different catalyst materials is recorded in Table 2. Moreover, this value of hydrogen evolution rate (22,340 mL/g·min) is higher than the rates achieved in the literature [58,61,62,63,64,65]. This remarkable development in the performance of the MoO_3_/S@g-C_3_N_4_ catalyst indicates its priority in the production of hydrogen from sodium borohydride.

## 4. Conclusions

The MoO_3_/g-C_3_N_4_ nanocomposites were functionalized for hydrogen production. Graphitic carbon nitride (g-C_3_N_4_)-based catalysis is prepared via thermal condensation of thiourea. The MoO_3_, g-C_3_N_4_, and MoO_3_/g-C_3_N_4_ nanocomposite catalysts were characterized by using XRD, FTIR, FESEM, STEM and spectrophotometer. When g-C_3_N_4_ and MoO_3_ were combined, the g-C_3_N_4_ was coated on the surface of MoO_3_ and turned to be positively charged. The lattice constant and the volume of MoO_3_/10-C_3_N_4_ was found to be the highest compared with MoO_3_, MoO_3_/20-C_3_N_4_, and MoO_3_/30C_3_N_4_. The nanocomposite sample MoO_3_/10%S@g-C_3_N_4_ showed a higher surface area and large pore volume. In addition, the combination of MoO_3_/10-C_3_N_4_ has a wide band gap energy of ~4.14 eV, whereas MoO_3_ has an energy band gap of 3.86 eV. The average nanocrystal size and microstrain for MoO_3_/10-C_3_N_4_ were found to be 23 nm and −0.042, respectively. The highest hydrogen production from NaBH_4_ hydrolysis ~22,340 mL/g·min was obtained from MoO_3_/10-C_3_N_4_ nanocomposites, while 18,421 mL/g·min was obtained from pure MoO_3_. Hydrogen production was increased when increasing the masses of MoO_3_/10-C_3_N_4_.

## Figures and Tables

**Table 1:** The XRD parameters of MoO_3_ coated with different concentrations of S@g-C_3_N_4_.

Sample	Lattice Constant (Å)			Volume (Å^3^)	Average Nanocrystals Size (nm)	Micro Strain
	a	b	c			
MoO_3_	3.96	13.86	3.69	202.5	30	0.00004
MoO_3_/10CN	3.96	13.92	3.69	203.4	23	−0.042
MoO_3_/20CN	3.95	13.84	3.69	201.7	28	−0.0037
MoO_3_/30CN	3.95	13.86	3.69	202.02	20	−0.030

**Table 2:** Comparison of hydrogen evolution rate for different catalysts at 25 °C.

Catalyst	Form	Hydrogen Evolution Rate (mL/g·min)	Ref.
Ni_2_P	Powder	3700	[62]
Ru/C	Powder	6060	[63]
g-C_3_N_4_-SiO_2_-N	Powder	11,400	[53]
Ru_5_Co/C	Powder	9360	[63]
B and O doped g-C_3_N_4_	Powder	11,600	[61]
Ru/NiO-Ni	Foam	6000	[64]
g-C_3_N_4_-TiO_2_-P	Powder	14,750	[55]
C-KOH-N	Powder	20,100	[65]
MoO_3_/10S@g-C_3_N_4_	Powder	22,340	This study

## Data Availability

Data available on request from the corresponding author.
